# Supplementary material for: Tailoring high-energy storage NaNbO3-based materials from antiferroelectric to relaxor states
Source: Nat Commun. 2023 Mar 18;14:1525. doi: 10.1038/s41467-023-37060-4 (PMC10024729; doi:10.1038/s41467-023-37060-4)
Supplement: Supplementary file 1 — Supplementary Information [file 41467_2023_37060_MOESM1_ESM.pdf]

Supplementary Information for

Tailoring high energy-storage $NaNbO_3$-based materials from antiferroelectric to relaxor states

Mao-Hua Zhang[1,9,*], Hui Ding[2], Sonja Egert[3], Changhao Zhao[1], Lorenzo Villa[4], Lovro Fulanović[1], Pedro B. Groszewicz[5], Gerd Buntkowsky[3], Hans-Joachim Kleebe[6], Karsten Albe[4], Andreas Klein[7], Jurij Koruza[1,8,*]

[1]Non-metallic Inorganic Materials, Department of Materials and Earth Sciences, Technical University of Darmstadt, Darmstadt 64287, Germany
[2]Advanced Electron Microscopy, Department of Materials and Earth Sciences, Technical University of Darmstadt, Darmstadt 64287, Germany
[3]Eduard Zintl Institute for Inorganic and Physical Chemistry, Technical University of Darmstadt, Darmstadt 64287, Germany
[4]Materials Modeling Division, Department of Materials and Earth Sciences, Technical University of Darmstadt, Darmstadt 64287, Germany
[5]Department of Radiation Science and Technology, Delft University of Technology, 2600AA Delft, Netherlands
[6]Institute of Applied Geosciences, Geomaterial Science, Technical University of Darmstadt, Darmstadt 64287, Germany
[7]Electronic Structure of Materials, Department of Materials and Earth Sciences, Technical University of Darmstadt, Darmstadt 64287, Germany
[8]Institute for Chemistry and Technology of Materials, Graz University of Technology, Graz 8010, Austria
[9]Current Address: Department of Materials Science and Engineering, The Pennsylvania State University, University Park, PA 16802, USA.

Corresponding authors:
jurij.koruza@tugraz.at (Jurij Koruza)
maohua.zhang.10@gmail.com (Mao-Hua Zhang)

Supplementary Fig. 1: Tolerance factor plots.
Supplementary Fig. 2: Backscattered electron microscopy (BSE) images.
Supplementary Fig. 3: X-ray diffraction (XRD) patterns of powder samples from crushed ceramic pellets.
Supplementary Figs. 4-7, 16-18: Polarization and strain hysteresis loops data.
Supplementary Figs. **8, 10**: High-resolution transmission electron microscopy results.
Supplementary Figs. **9, 11, 12**: High-energy XRD results.
Supplementary Figs. 13 & 19: Schematics of crystallographic structures.
Supplementary Figs. 14 & 15: Solid-state ^{23}Na nuclear magnetic resonance (NMR) spectroscopy results.
Supplementary Fig. 20: Density functional theory (DFT) calculations.
Supplementary Table. 1: NMR parameters.
Supplementary Tables. 2-6: Structural parameters.
Supplementary Table. 7: Electrical properties.

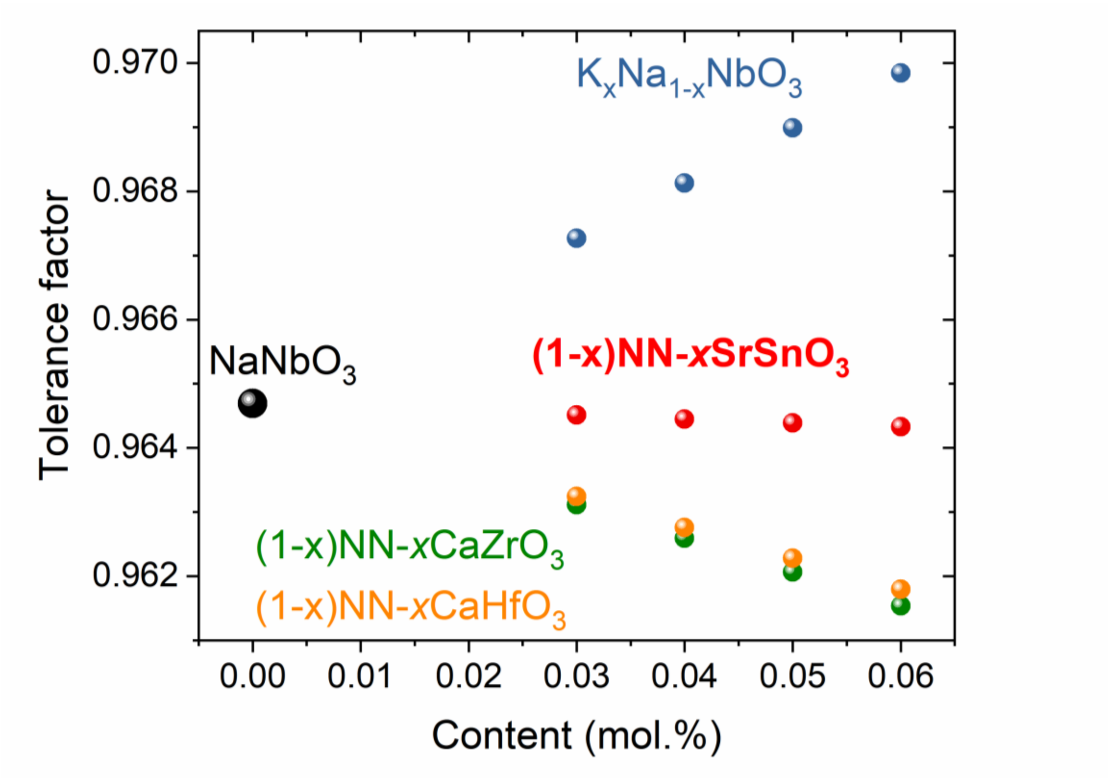

Supplementary Fig. 1. Tolerance factor of the $NaNbO_3$-$SrSnO_3$ solid solution and other representative systems. The ionic radii data [Sr^{2+} (1.44 Å, CN=12), Sn^{4+} (0.69 Å, CN=6), Na^{+} (1.39 Å, CN=12), and Nb^{5+} (0.64 Å, CN=6)] are taken from the Shannon´s Database of Ionic Radii (Shannon, Acta Crystallogr., **1976**, 32, 751-767). The tolerance factor of pure $NaNbO_3$ is 0.965 and remains almost unchanged for NN5SS (0.964).

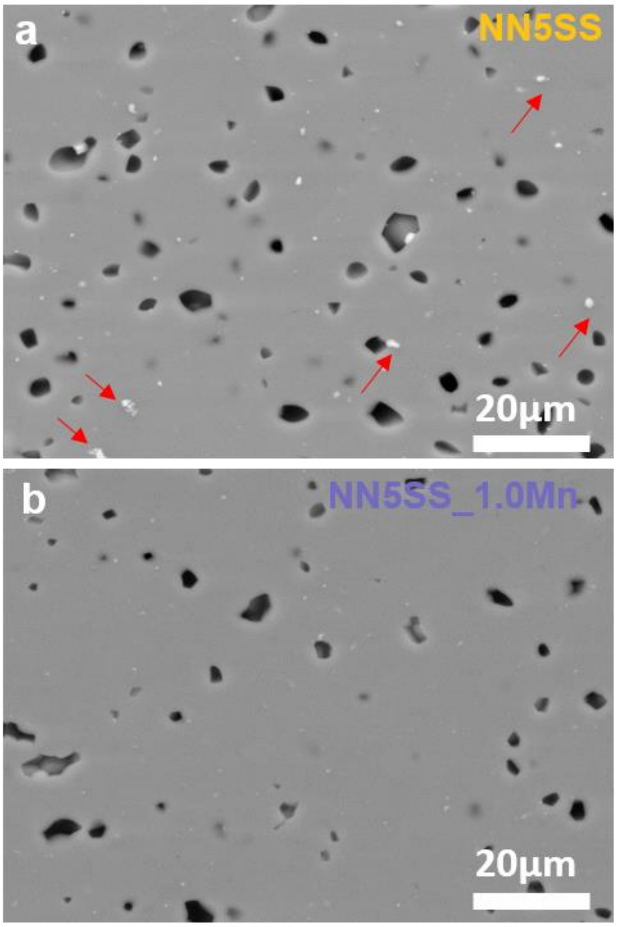

Supplementary Fig. 2. Backscattered electron microscopy (BSE) images of polished **a** NN5SS and **b** NN5SS_1.0Mn ceramic samples. SnO_2 inclusions are highlighted by red arrows. The amount of SnO_2 inclusions is significantly reduced after Mn addition.

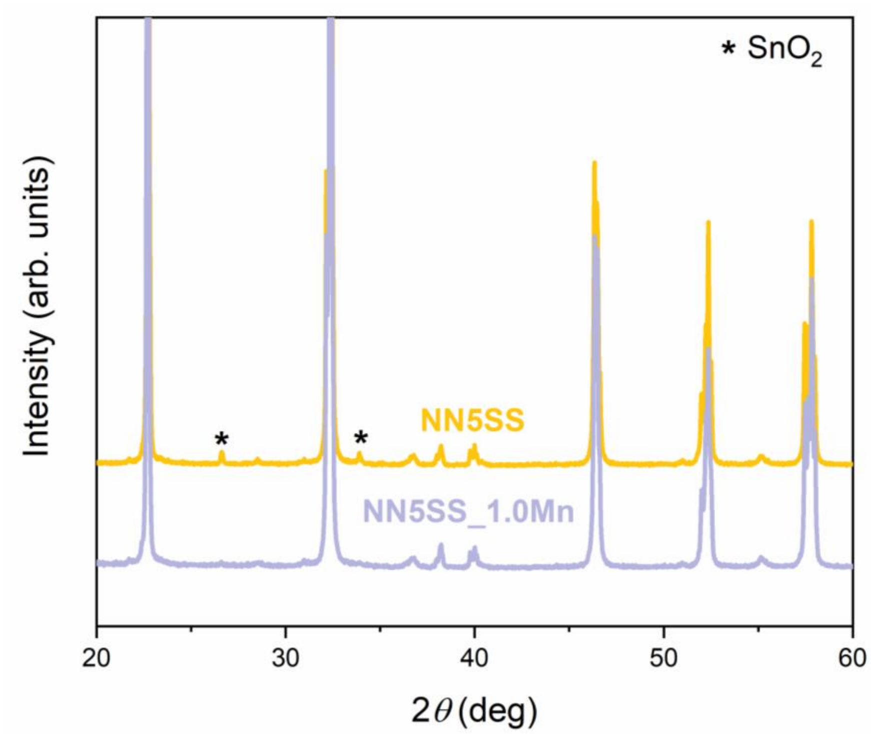

Supplementary Fig. 3. X-ray diffraction (XRD) patterns of NN5SS and NN5SS_1.0Mn powder samples from crushed ceramic pellets. The reflections of SnO_2 inclusions are visible in the NN5SS sample, but their intensity is much lower in the Mn-modified NN5SS_1.0Mn sample.

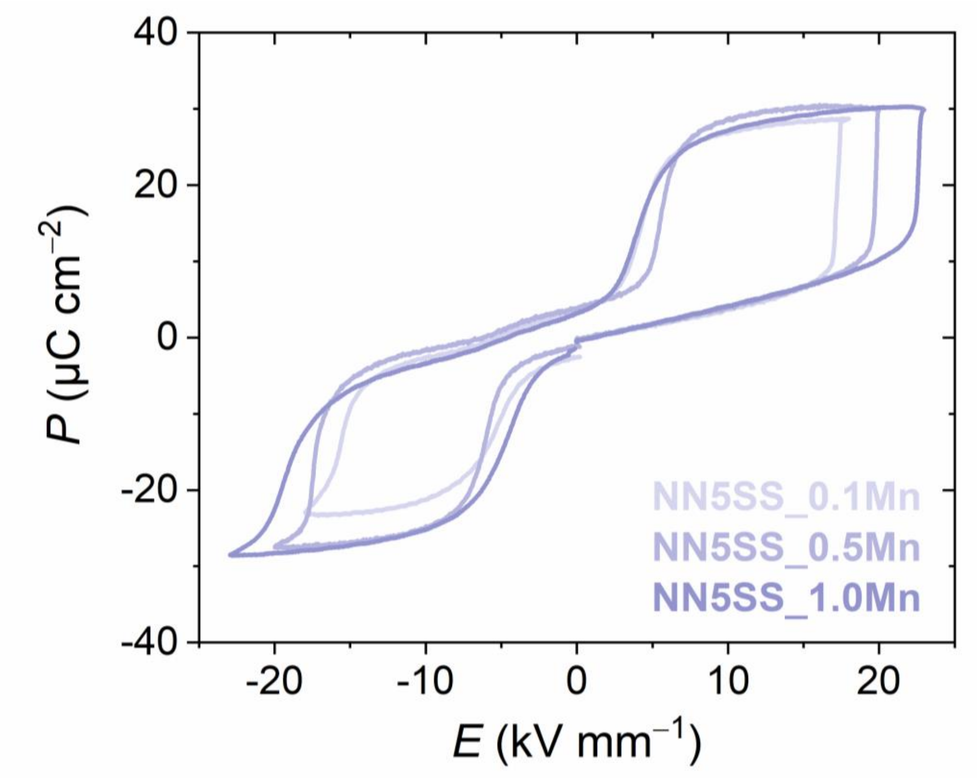

Supplementary Fig. 4. Polarization hysteresis loops of Mn-modified NN5SS_0.1Mn, NN5SS_0.5Mn, and NN5SS_1.0Mn samples during the first electric field cycle, measured at 1 Hz. The critical field required to trigger the transition increases with increasing Mn content.

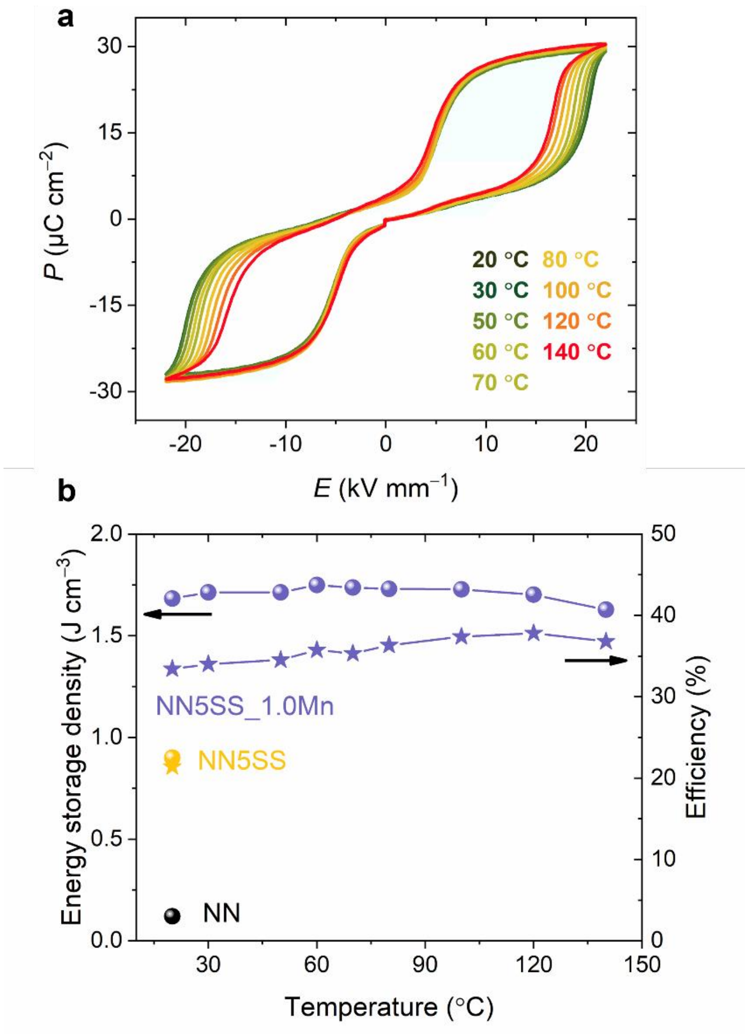

Supplementary Fig. 5. **a** Temperature-dependent polarization hysteresis loops of NN5SS_1.0Mn sample, measured at 1 Hz and **b** the temperature dependence of energy-storage density and efficiency calculated from the loops. The energy storage density of $NaNbO_3$ (NN) and the energy storage density and efficiency of 0.95$NaNbO_3$-0.05$SrSnO_3$ (NN5SS) are labeled. Note that due to the experimental limitations, the maximal measured temperature was 140 °C.

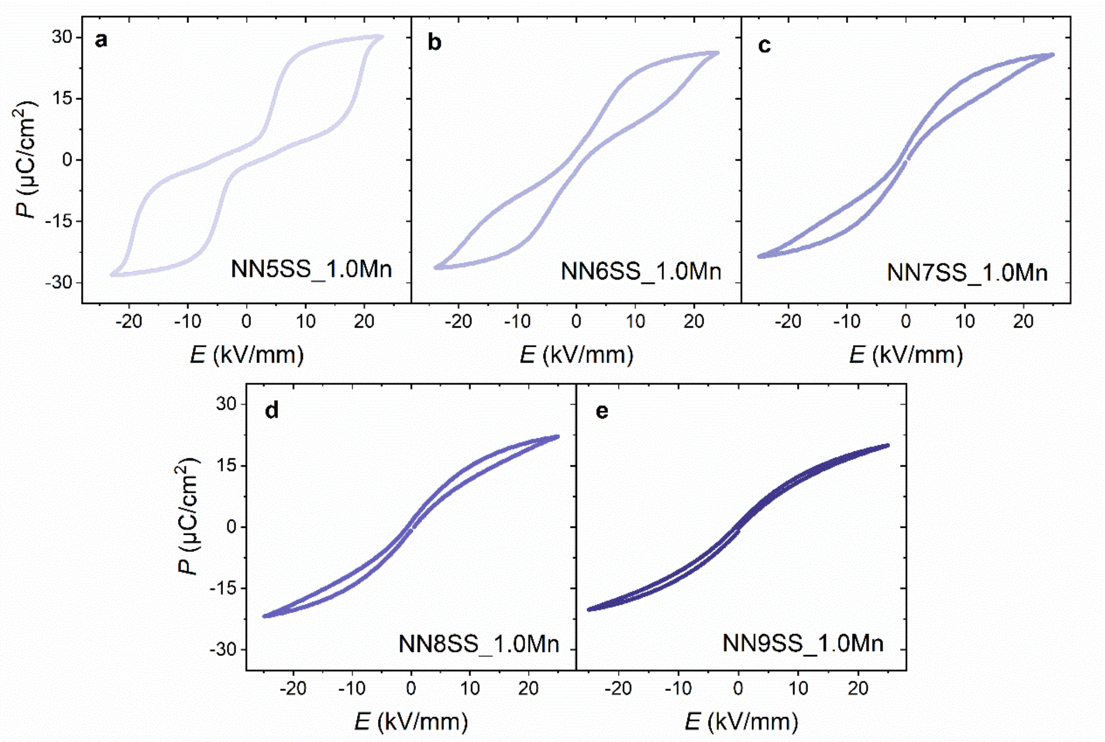

Supplementary Fig. 6. Polarization hysteresis loops of **a** NN5SS_1.0Mn, **b** NN6SS_1.0Mn, **c** NN7SS_1.0Mn, **d** NN8SS_1.0Mn, **e** NN9SS_1.0Mn samples, measured at room temperature and 1 Hz.

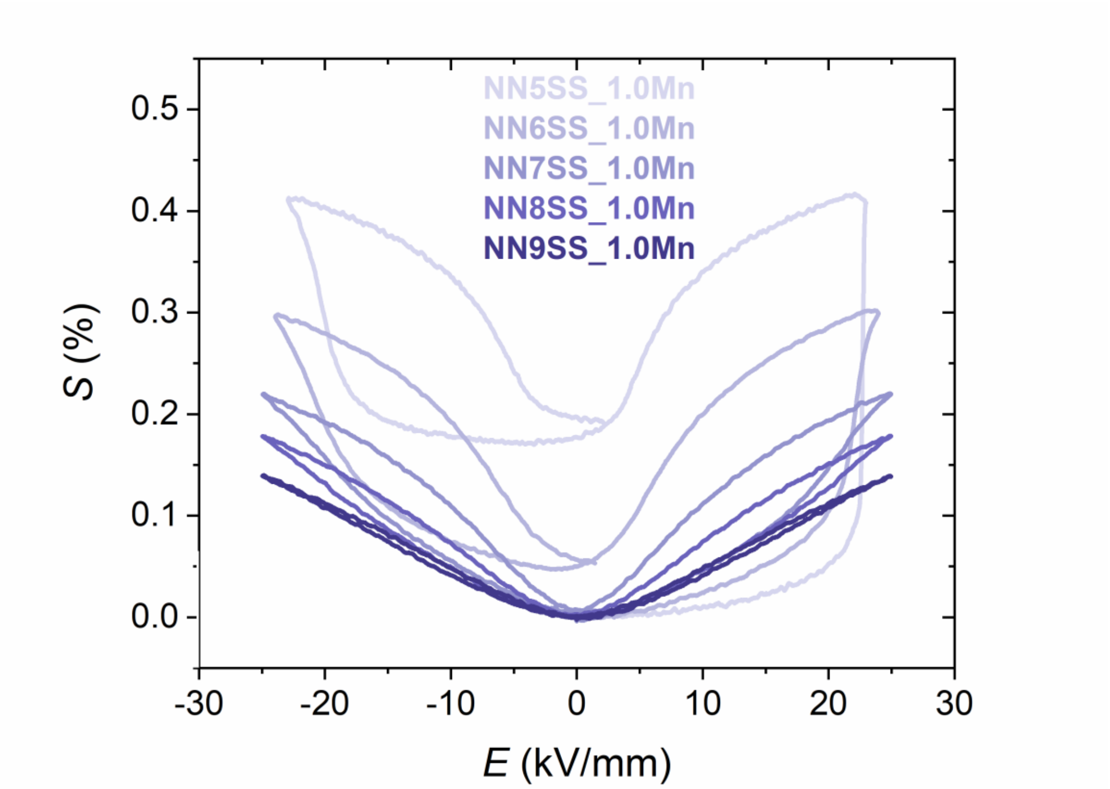

Supplementary Fig. 7. Strain hysteresis loop of the NN5SS_1.0Mn, NN6SS_1.0Mn, NN7SS_1.0Mn, NN8SS_1.0Mn, and NN9SS_1.0Mn samples, recorded along the longitudinal direction in the first electric field cycle.

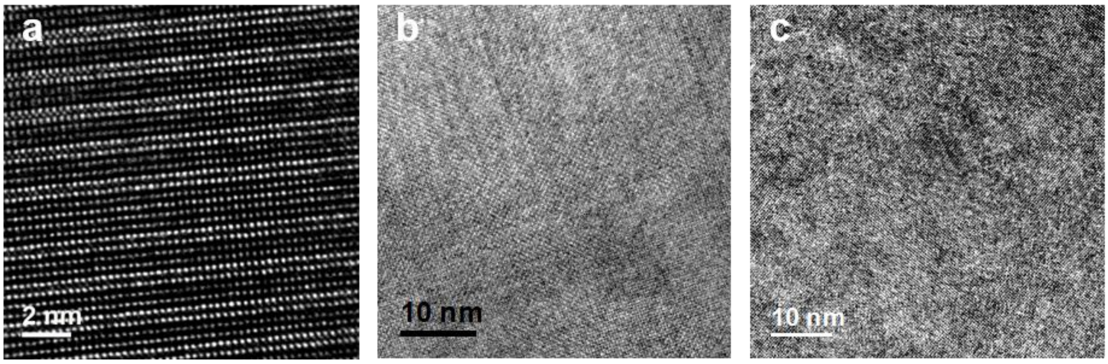

Supplementary Fig. 8. High-resolution transmission electron microscopy (HRTEM) images of **a** NN5SS_1.0Mn, **b** NN7SS_1.0Mn, and **c** NN9SS_1.0Mn.

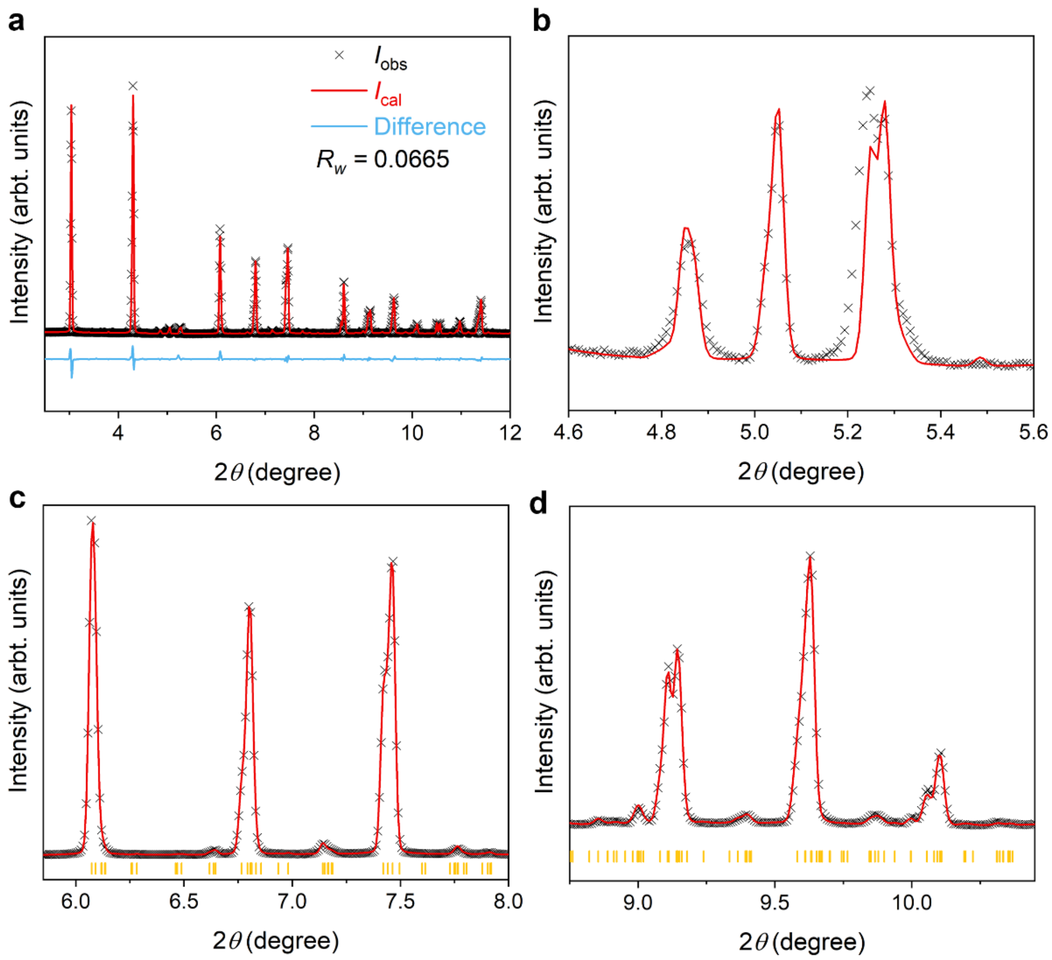

Supplementary Fig. 9. Rietveld refinement of the high-energy XRD pattern of the NN5SS_1.0Mn sample using the *Pbcm* structural model. **a** Full pattern, magnified views of **b** representative superlattice reflections and **c**-**d** high-angle main reflections. Reflections associated with the *Pbcm* structure are shown by the yellow tick marks.

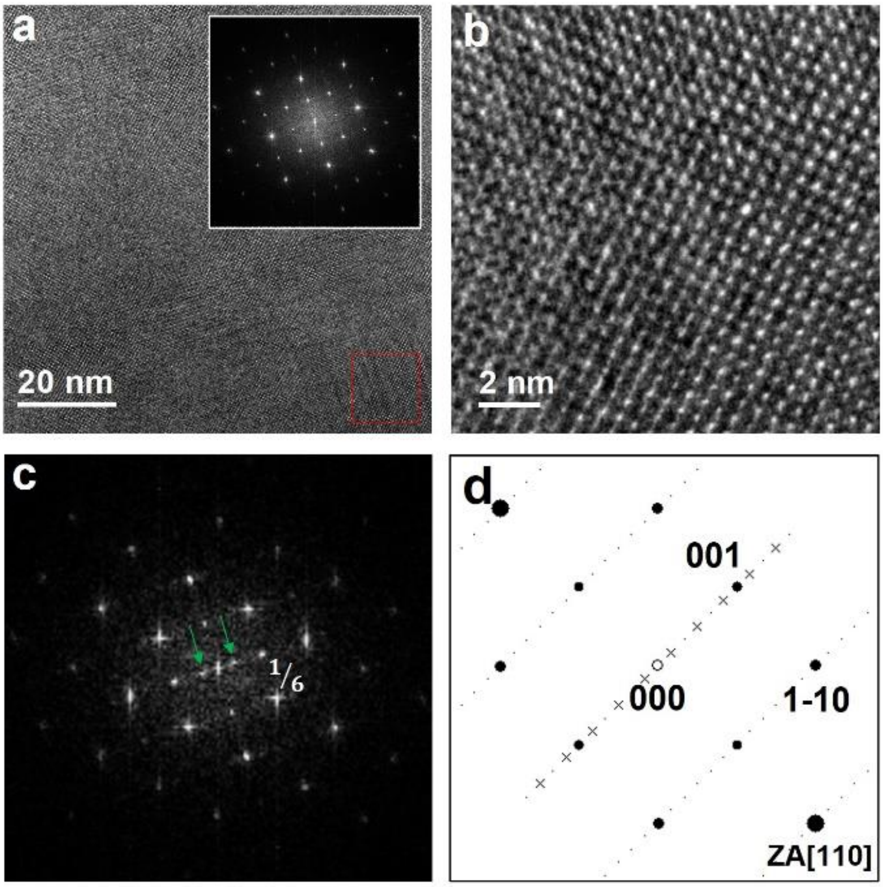

Supplementary Fig. 10: **a** TEM micrograph of NN7SS_1.0Mn with a fast Fourier transform (FFT) pattern inset. **b** The enlarged graph from the red square in a. **c** FFT pattern of b, where the characteristic $^1/_6$ superlattice reflections are marked by the green arrows. **d** Simulated electron diffraction patterns in the [110] zone axis using the *Pbnm* space group with the software CrysTBox. Note that the characteristic $^1/_6$ superlattice reflections can only be observed in **c** (at a local scale), while they are not present when recorded from a larger area as seen in **a**.

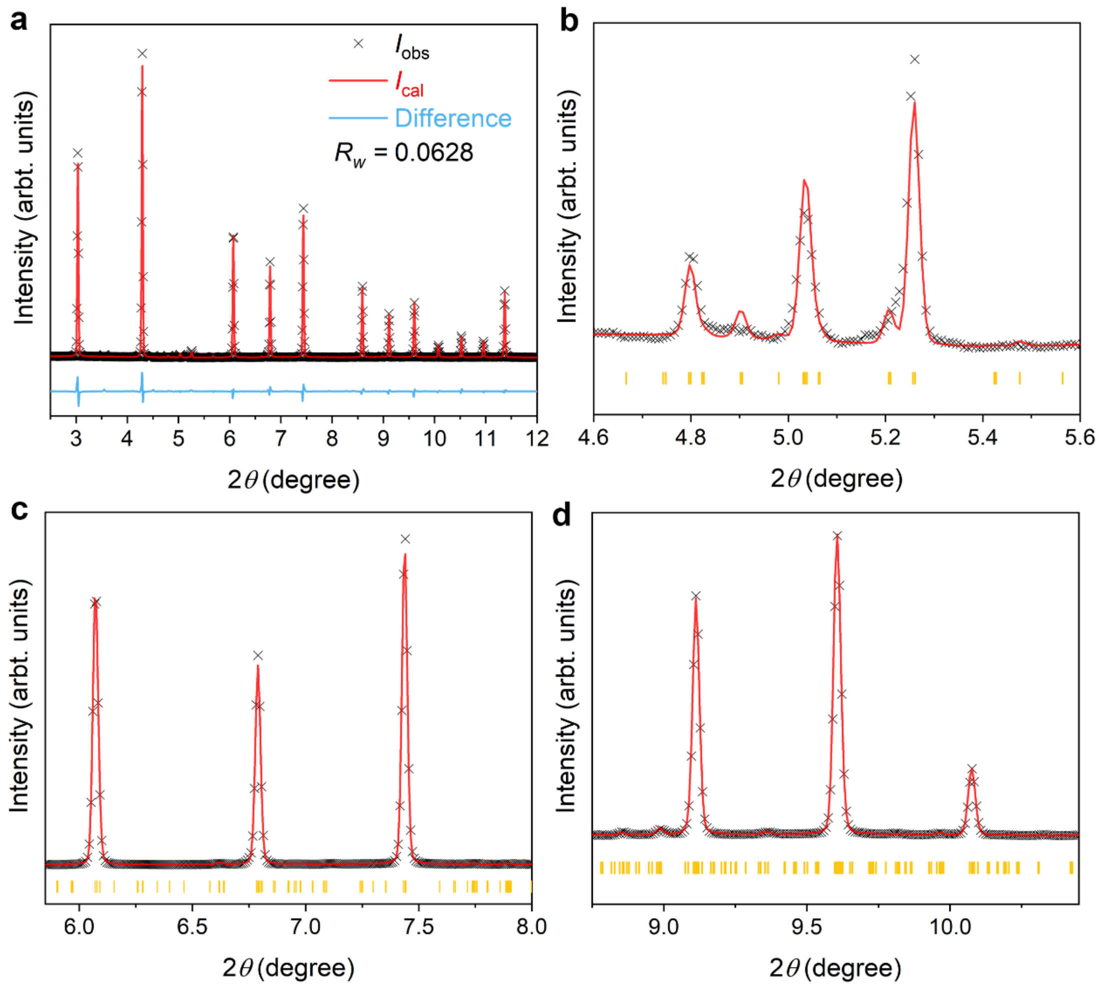

Supplementary Fig. 11. Rietveld refinement of the high-energy XRD pattern of the NN9SS_1.0Mn sample using the *Pbnm* structural model. **a** Full pattern, magnified views of **b** representative superlattice reflections and **c**-**d** high-angle main reflections. Reflections associated with the *Pbnm* structure are shown by the yellow tick marks. It can be seen that the main reflections can be well described by the *Pbnm* model.

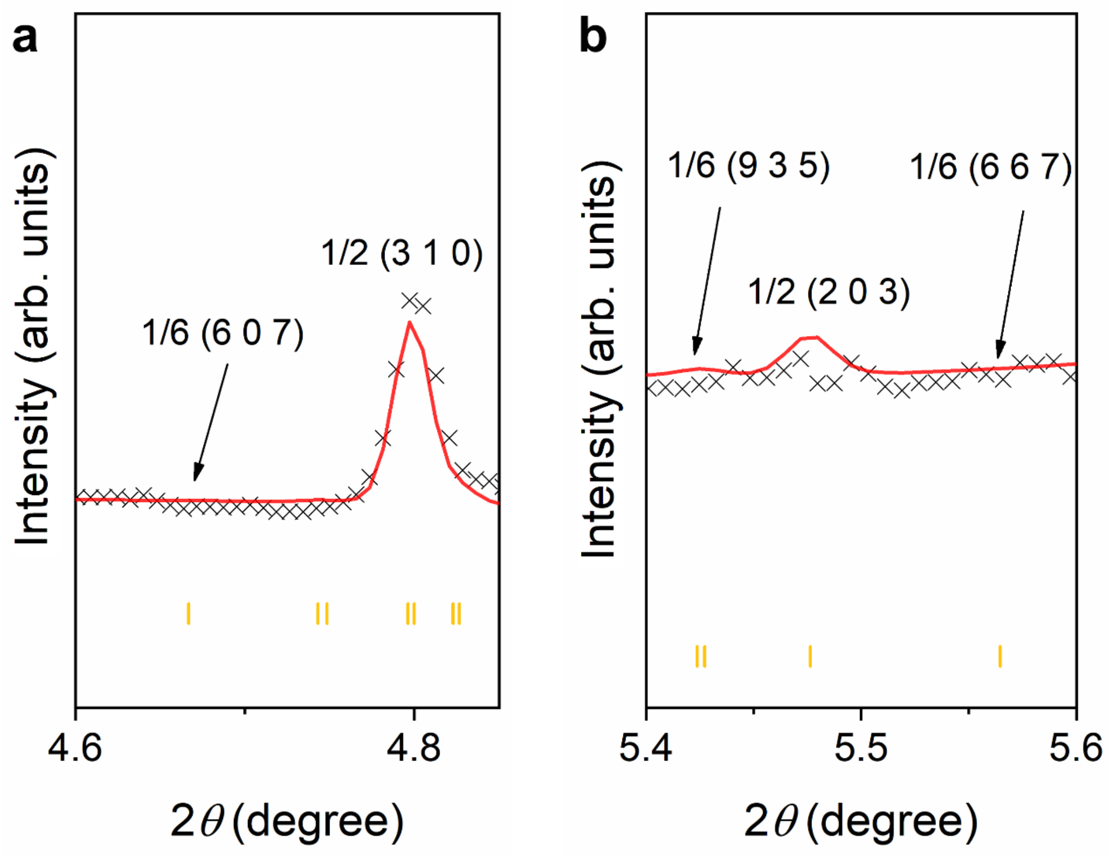

Supplementary Fig. 12. **a-b** Selected 2θ ranges of the high-energy XRD pattern of the NN9SS_1.0Mn sample, showing that the $^1/_6$ superlattice reflections are not satisfactorily described by the *Pbnm* model. Reflections associated with the *Pbnm* structure are shown by the yellow tick marks.

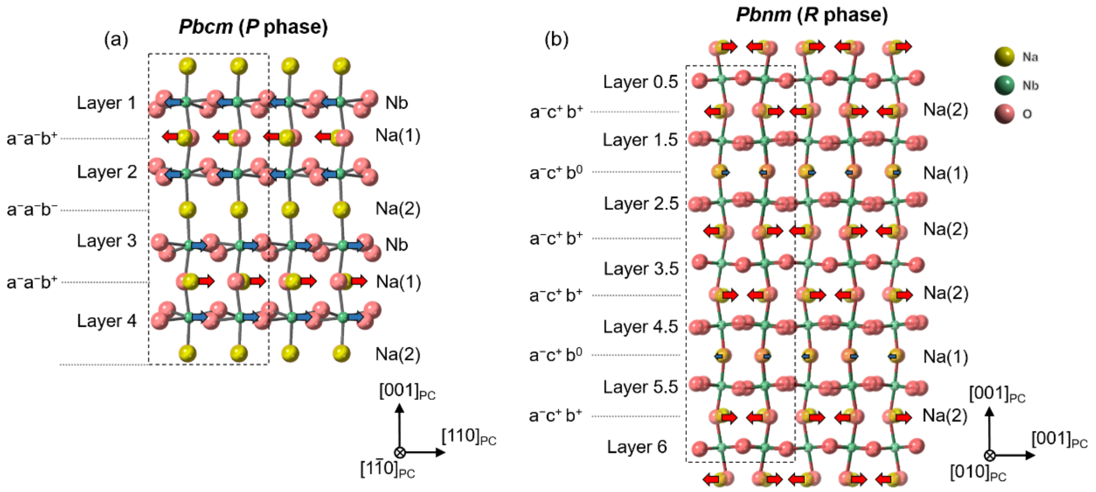

Supplementary Fig. 13. Crystallographic structures of **a** the *P* phase (*Pbcm* space group) and **b** the *R* phase (*Pbnm* space group), viewed along the $[1\bar{1}0]_{PC}$ direction and $[010]_{PC}$ direction, respectively. The unit cells are highlighted by the dashed lines and the tilting systems between the adjacent octahedral layers are marked. The *P* phase is characterized by ¼ superlattice reflections due to the quadrupling of the aristotype perovskite structure along the $[001]_{PC}$ direction. In contrast, the unit cell of the *R* phase corresponds to a $\sqrt{2} \times \sqrt{2} \times 6$ superlattice of the aristotype perovskite structure, giving rise to 1/6 superlattice reflections. Despite the presence of antiferrodistortion in the *R* phase, no double polarization hysteresis loops have been reported. The investigated NN9SS_1.0Mn sample (in *R*' phase) does not have long-range antiferroelectric order that would otherwise lead to the well-defined 1/6 superlattice reflections.

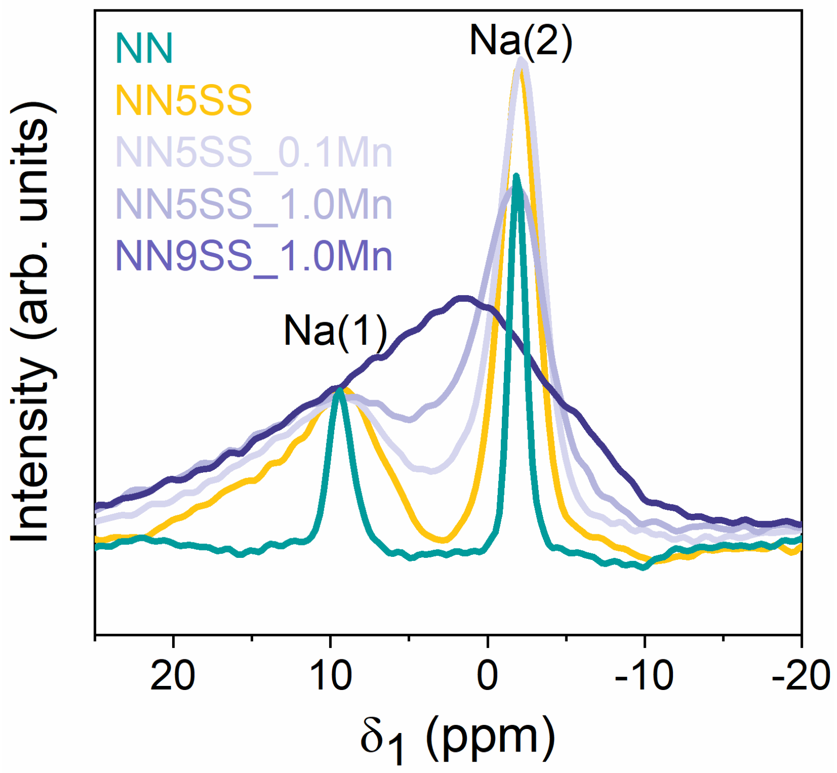

Supplementary Fig. 14. Summation of the NMR signals of NN, NN5SS, NN5SS_0.1Mn, NN5SS_1.0Mn, and NN9SS_1.0Mn samples along the indirect dimension δ_1.

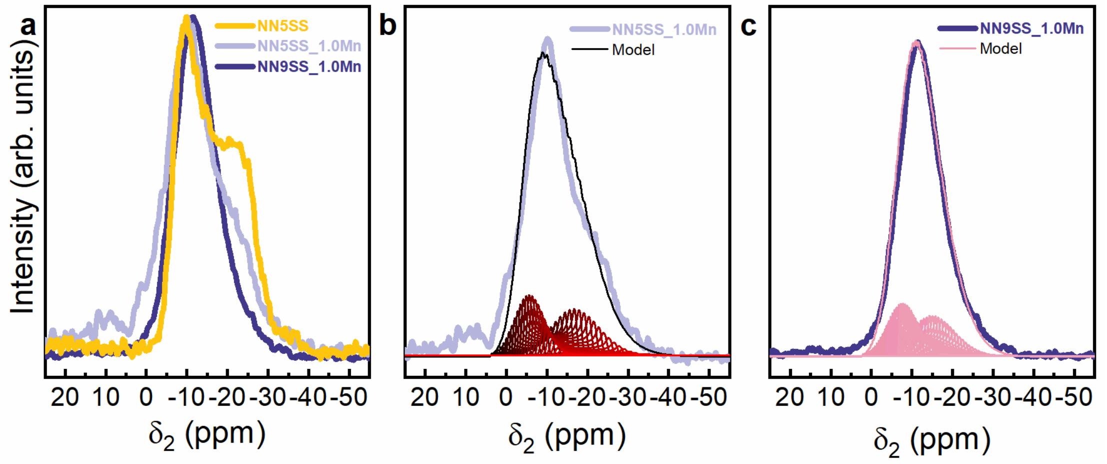

Supplementary Fig. 15. **a** Comparison of the projections of the Na(2) signals of NN5SS and NN5SS_1.0Mn and the total signal of NN9SS_1.0Mn onto the direct axis of the STMAS spectrum, revealing the characteristic quadrupolar broadened line shapes. Across the range of samples, the right flank becomes increasingly narrower and attenuated. **B** Line shape simulation of NN5SS_1.0Mn with a set of 30 dependent lines and **c** line shape simulation of NN9SS_1.0Mn with a set of 40 dependent lines, yielding the parameters reported in Supplementary Table 1. Simulations for both compounds were carried out assuming positively correlated, but otherwise independent distributions of the quadrupolar coupling constant and the isotropic chemical shift, which have previously shown to approximate the Na(2) line shape of NN5SS well (Zhang *et al.*, Chem. Mater. **2021**, 33, 266).

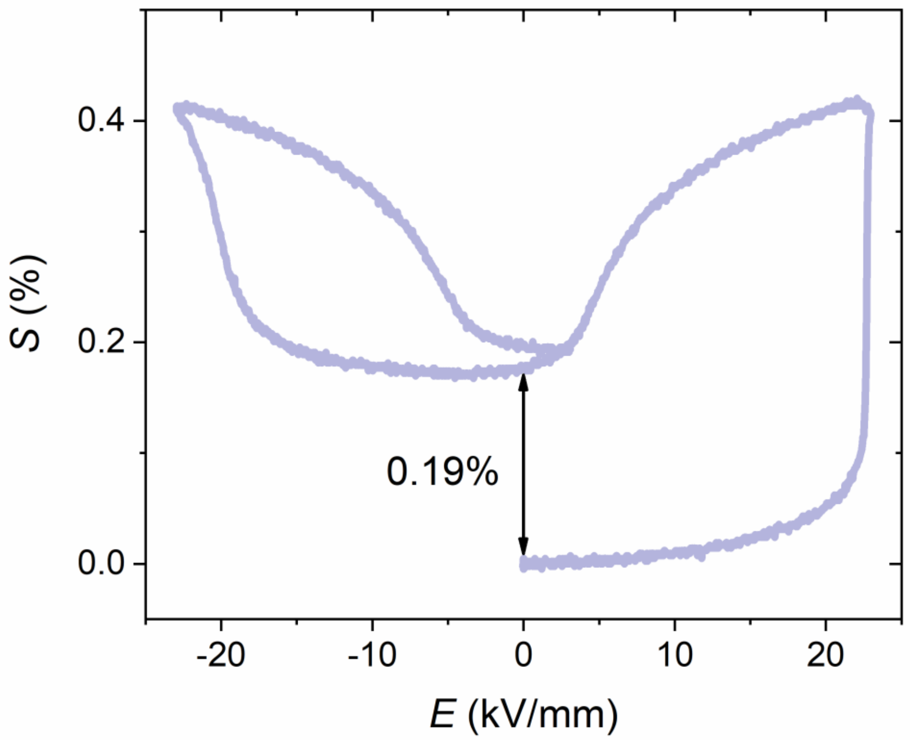

Supplementary Fig. 16. Strain hysteresis loop of the NN5SS_1.0Mn sample, recorded along the longitudinal direction in the first electrical cycle. A remanent strain of 0.19% is observed.

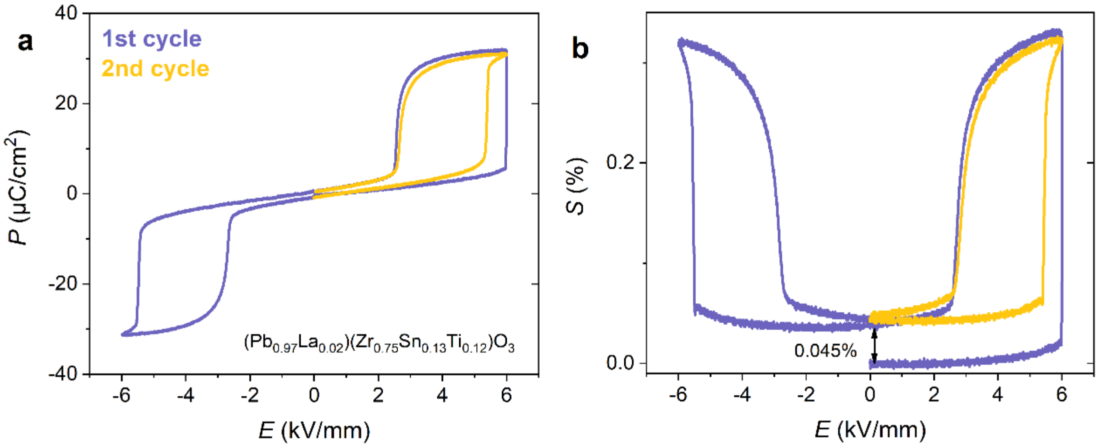

Supplementary Fig. 17. **a** Polarization and **b** strain hysteresis loop of a $(Pb_{0.97}La_{0.02})(Zr_{0.75}Sn_{0.13}Ti_{0.12})O_3$ ceramic sample in the 1st and 2nd electrical cycle.

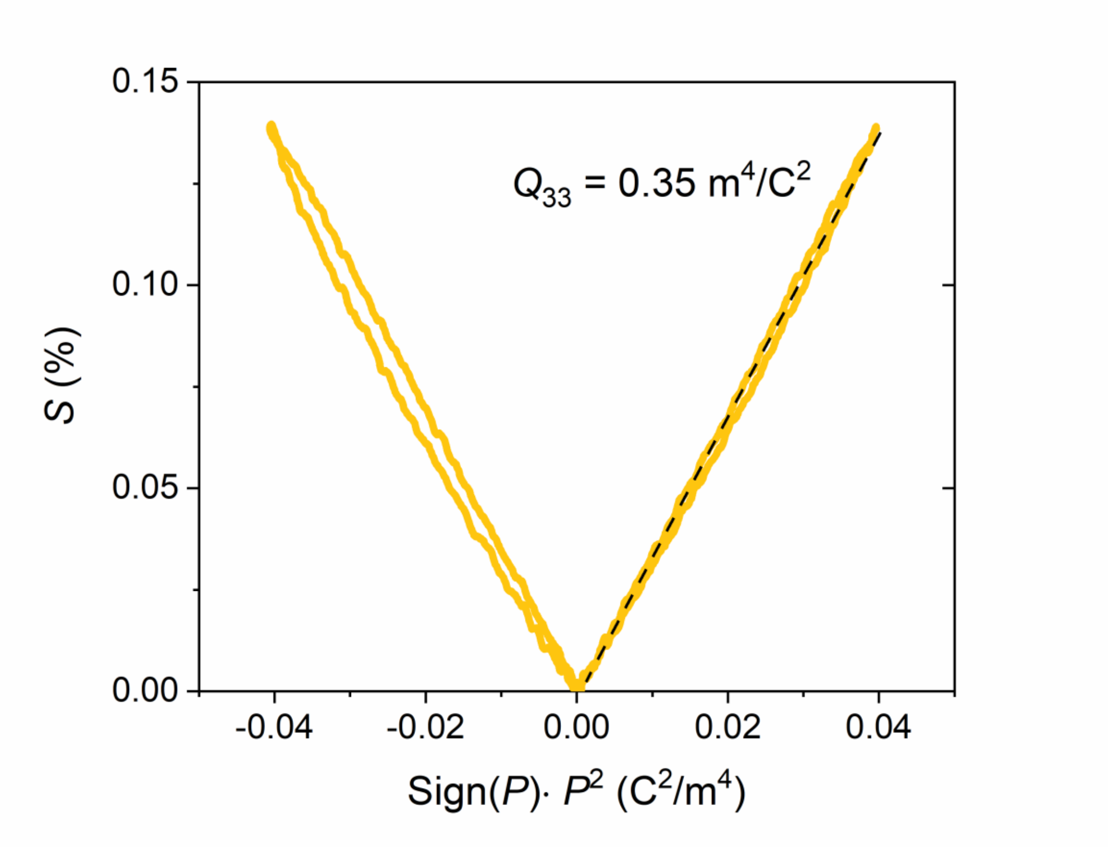

Supplementary Fig. 18. Strain-polarization^2 (S-Sign(P)·P^2) loop of NN9SS_1.0Mn sample. The fitted electrostrictive coefficient (0.35 m^4/C^2) is highlighted.

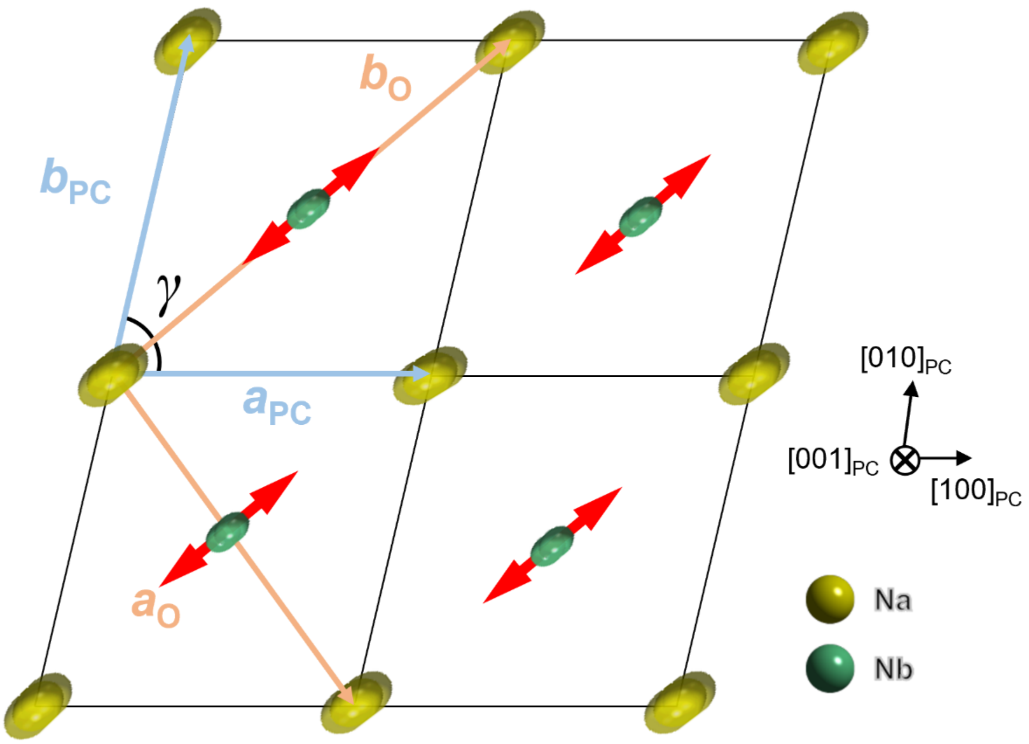

Supplementary Fig. 19. A schematic of the crystallographic structure of the $NaNbO_3$-based materials with the *Pbcm* space group along the $[001]_{PC}$ direction. The orientation of the double-headed arrow represents the direction of the antiparallel atomic displacement of Nb atoms.

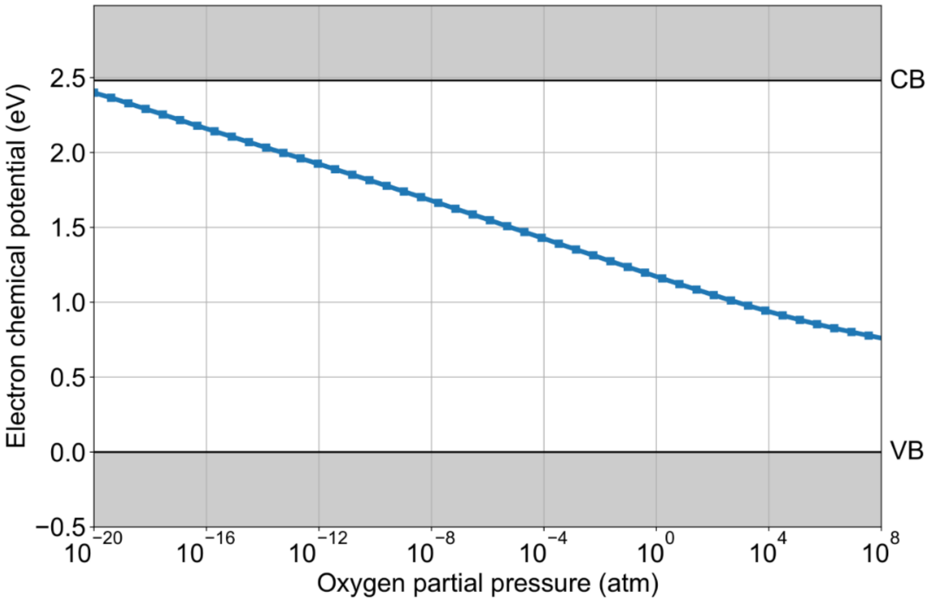

Supplementary Fig. 20. Fermi level as a function of the oxygen partial pressure.

Supplementary Table. 1. Comparison of quadrupolar coupling constants C_Q, isotropic chemical shifts δ_{iso}, and distribution widths Δ thereof for the investigated materials. The data are obtained from line shape simulations as depicted in Figure R1 based on negatively correlated Gaussian distributions of both NMR parameters; values for NN_5SS have previously been reported in Zhang *et al*., Chem. Mater. **2021**, 33, 266.

Composition	C_Q (kHz)	ΔC_Q (kHz)	δ_{iso} (ppm)	$\Delta\delta_{iso}$ (ppm)
NN5SS	1900	130	-2.8	1.2
NN5SS_1.0Mn	1600	160	-2.4	2.8
NN9SS_1.0Mn	1300	140	-5.9	3.2

Supplementary Table 2. The refined cell parameters of NN, NN5SS, NN5SS_0.1Mn, NN5SS_0.5Mn, NN5SS_1.0Mn, and NN9SS_1.0Mn samples.

	a (Å)	*b* (Å)	*c* (Å)	*V* ($Å^3$)	Space Group	R_w
NN	5.50346 (10)	5.56652 (10)	15.53871 (24)	476.031 (10)	*Pbcm*	0.0524
NN5SS	5.51535 (7)	5.56344 (6)	15.61913 (21)	479.263 (7)	*Pbcm*	0.0892
NN5SS_0.1Mn	5.51512 (12)	5.56325 (11)	15.6168 (4)	479.154 (11)	*Pbcm*	0.0623
NN5SS_0.5Mn	5.51441 (12)	5.56248 (11)	15.6166 (4)	479.021 (11)	*Pbcm*	0.0622
NN5SS_1.0Mn	5.51354 (13)	5.56140 (12)	15.6151 (4)	478.805 (12)	*Pbcm*	0.0665
NN9SS_1.0Mn	5.53661 (16)	5.54415 (12)	23.4711 (5)	720.465 (9)	*Pbnm*	0.0628

Supplementary Table 3. Refined structural parameters of NN5SS_0.1Mn with *Pbcm* model.

	x (Å)	*y* (Å)	*z* (Å)	U_{iso}
A1-site	0.2460(9)	0.25	0	0.0019(5)
A2-site	0.2460(9)	0.2227(9)	0.25	0.0019(5)
B-site	0.2513(2)	0.734(0)	0.1247(6)	0.0010(9)
O1-site	0.6670(0)	0.25	0	0.0021(0)
O2-site	0.2119(7)	0.7588(5)	0.25	0.0021(0)
O3-site	0.4751(3)	0.4618(6)	0.1494(3)	0.0021(0)
O4-site	0.0286(4)	0.0344(8)	0.1132(8)	0.0021(0)
R_{wp}		6.23		
GOF (χ^2)		3.17		

Supplementary Table 4. Refined structural parameters of NN5SS_0.5Mn with *Pbcm* model.

	x (Å)	y (Å)	z (Å)	U_{iso}
A1-site	0.2422(7)	0.25	0	0.0008(7)
A2-site	0.2422(7)	0.2207(9)	0.25	0.0008(7)
B-site	0.2513(0)	0.7347(8)	0.1247(8)	0.0012(0)
O1-site	0.6621(4)	0.25	0	0.0000(8)
O2-site	0.2108(4)	0.7596(7)	0.25	0.0000(8)
O3-site	0.4774(0)	0.4592(2)	0.1491(5)	0.0000(8)
O4-site	0.0257(8)	0.0343(5)	0.1130(2)	0.0000(8)
R_{wp}		6.22		
GOF (χ^2)		3.13		

Supplementary Table 5. Refined structural parameters of NN5SS_1.0Mn with *Pbcm* model.

	x (Å)	y (Å)	z (Å)	U_{iso}
A1-site	0.2454(2)	0.25	0	0.0008(7)
A2-site	0.2454(2)	0.2166(3)	0.25	0.0008(7)
B-site	0.2512(2)	0.7360(3)	0.1247(6)	0.0012(0)
O1-site	0.6608(6)	0.25	0	0.0000(8)
O2-site	0.2110(7)	0.7585(8)	0.25	0.0000(8)
O3-site	0.4777(9)	0.4504(3)	0.1508(5)	0.0000(8)
O4-site	0.0258(6)	0.0340(8)	0.1129(3)	0.0000(8)
R_{wp}		6.65		
GOF (χ^2)		3.32		

Supplementary Table 6. Refined structural parameters of NN9SS_1.0Mn with *Pbnm* model.

	x (Å)	y (Å)	z (Å)	U_{iso}
A1-site	0.5071(9)	0.5020(0)	0.25	0.0129(7)
A2-site	0.9984(6)	0.0018(7)	0.0836(5)	0.0129(7)
B1-site	0.5	0	0	0.0044(6)
B2-site	0.4983(2)	0.0237(7)	0.3338(1)	0.0044(6)
O1-site	0.5457(5)	0.4870(0)	0.25	0.0191(5)
O2-site	0.0601(5)	0.2417(1)	0.0889(5)	0.0339(9)
O3-site	0.7393(4)	0.3005(1)	0.0087(2)	0.0279(9)
O4-site	0.7170(1)	0.3413(6)	0.3413(6)	0.0057(4)
O5-site	0.7115(5)	0.2832(9)	0.6700(0)	0.0072(6)
R_{wp}		6.28		
GOF (χ^2)		2.89		

Supplementary Table 7. Electrical Properties of NN5SS, NN5SS_1.0Mn, and NN9SS_1.0Mn samples.

	NN5SS	NN5SS_1.0Mn	NN9SS_1.0Mn
ε (RT, @10 kHz)	345	325	1444
P_r (μC cm^{-2})	11.0	3.2	0.8
P_m (μC cm^{-2})	31.1	30.1	20.0
	@ 16 kV mm^{-1}	@ 23 kV mm^{-1}	@25 kV mm^{-1}
W_{stor} (J cm^{-3})	4.2	5.2	2.0
W_{rec} (J cm^{-3})	0.90	1.70	1.75
η (%)	21	33	90

ε: dielectric permittivity, *P*r: remanent polarization, P_m: polarization at the maximum electric field, W_{stor}: storage energy density, W_{rec}: recoverable energy density and η: energy-storage efficiency (W_{stor}/W_{rec}).
